# No Accumulation of Transposable Elements in Asexual Arthropods

**DOI:** 10.1093/molbev/msv261

**Published:** 2015-11-11

**Authors:** Jens Bast, Ina Schaefer, Tanja Schwander, Mark Maraun, Stefan Scheu, Ken Kraaijeveld

**Affiliations:** ^1^Department of Ecology and Evolution, University of Lausanne, Lausanne, Switzerland; ^2^J.F. Blumenbach Institute of Zoology and Anthropology, Georg August University Goettingen, Goettingen, Germany; ^3^Department of Ecological Science, VU University Amsterdam, Amsterdam, The Netherlands; ^4^Leiden Genome Technology Center, Department of Human genetics, Leiden University Medical Center, Leiden, The Netherlands

**Keywords:** evolution of sex, transposable elements, parthenogenesis, oribatid mites, genetic load, TE accumulation

## Abstract

Transposable elements (TEs) and other repetitive DNA can accumulate in the absence of recombination, a process contributing to the degeneration of Y-chromosomes and other nonrecombining genome portions. A similar accumulation of repetitive DNA is expected for asexually reproducing species, given their entire genome is effectively nonrecombining. We tested this expectation by comparing the whole-genome TE loads of five asexual arthropod lineages and their sexual relatives, including asexual and sexual lineages of crustaceans (*Daphnia* water fleas), insects (*Leptopilina* wasps), and mites (*Oribatida*). Surprisingly, there was no evidence for increased TE load in genomes of asexual as compared to sexual lineages, neither for all classes of repetitive elements combined nor for specific TE families. Our study therefore suggests that nonrecombining genomes do not accumulate TEs like nonrecombining genomic regions of sexual lineages. Even if a slight but undetected increase of TEs were caused by asexual reproduction, it appears to be negligible compared to variance between species caused by processes unrelated to reproductive mode. It remains to be determined if molecular mechanisms underlying genome regulation in asexuals hamper TE activity. Alternatively, the differences in TE dynamics between nonrecombining genomes in asexual lineages versus nonrecombining genome portions in sexual species might stem from selection for benign TEs in asexual lineages because of the lack of genetic conflict between TEs and their hosts and/or because asexual lineages may only arise from sexual ancestors with particularly low TE loads.

## Introduction

Genetic linkage in the absence of recombination couples the fates of different mutations and thereby decreases the efficacy of natural selection (Muller 1964; Hill and Robertson 1966; Nuzhdin and Petrov 2003). An important consequence of the reduced efficacy of selection is the accumulation of deleterious mutations and repetitive DNA in the form of transposable elements (TEs), a process well documented for the nonrecombining Y (and W) sex chromosomes (Bachtrog 2013) and other nonrecombining genome portions (Lynch and Blanchard 1998; Schwander et al. 2014; Leung et al. 2015). Repetitive DNA enrichment in the absence of recombination is substantial and occurs surprisingly rapidly. For example, 19% of the neo-Y chromosome in the fruitfly *Drosophila miranda* consists of repetitive sequences, compared to only 1% of the neo-X (Bachtrog et al. 2008). The enrichment of repetitive DNA on the neo-Y would have occurred within less than 1 Ma—the estimated time frame for the origin of the neo-Y from a former autosome. Similar dynamics are known from other nonrecombining chromosomes: Muller F elements in *Drosophila* are repeat enriched 5–10-fold relative to recombining genome portions, whereby repeats constitute up to 50% of these elements (Leung et al. 2015). The rapid proliferation of TEs in the absence of recombination stems from their ability to self-replicate, via different mechanisms, to new positions in the genome, independently of the host's cell cycle (Hickey 1982; Burt and Trivers 2006).

Similar to nonrecombining genome portions, genomes of asexually reproducing animals are expected to accumulate TEs. The accumulation is anticipated to be especially massive in this case, given the entire genome is nonrecombining. Thus, TE accumulation has been considered a major factor generating lineage-level selection for sex in natural populations, driving asexual lineage decay and eventual extinction (Nuzhdin and Petrov 2003; Arkhipova and Meselson 2005). Relative to other types of deleterious mutations, TEs are especially likely to cause asexual lineage decay because of the severe phenotypic effects associated with TE activity (Burt and Trivers 2006) and their high mutation rates (generated by 10^−^^4^–10^−^^6^ transpositions per element per generation) that are orders of magnitude higher than point mutation rates (10^−^^8^–10^−^^9^; Arkhipova and Meselson 2005; Keightley et al. 2014). TE-driven decay of asexual lineages could thus explain why obligate asexuality is rare among eukaryotes, and why asexual lineages tend to be restricted to the tips of the tree of life (Arkhipova and Meselson 2005).

In spite of the potential importance of TEs for explaining the rarity of asexual lineages on the tree of life, the effect of genome-wide recombination loss on TE dynamics in natural populations remains thus far unknown. A land-mark study reported that genomes of ancient asexual bdelloid rotifers harbor fewer TEs than most animals (2.2% of their genomes consist of TEs; Arkhipova and Meselson 2000; Flot et al. 2013). This finding was consistent with the idea that only in the absence of active TEs would asexual lineages be able to persist over evolutionary time scales (millions of years). However, recent findings indicate that bdelloid rotifers are not fully asexual but engage in noncanonical forms of sexual reproduction (Signorovitch et al. 2015). Furthermore, because no sexual relatives were available for comparison, it is not possible to infer whether low TE content in bdelloids is linked to their reproductive mode or characteristic of rotifers (sexual and asexual linages) in general.

Because reproductive mode is a lineage-level trait, establishing links between asexuality and TE dynamics requires replicated comparisons of multiple, independently derived asexuals with sexual relatives. This is especially relevant in the context of TE dynamics, given TE contents are highly lineage specific, with extensive variation between populations and taxa (Clark et al. 2007; Kofler et al. 2012). Finally, because TEs are characterized by a diversity of distinct classes and (super)families that vary in their transposition mechanisms (Wicker et al. 2007), only whole-genome data quantifying all types of elements are useful for testing the effect of reproductive mode on TE dynamics (Rho et al. 2010; Schaack, Choi, et al. 2010; Schaack, Pritham, et al. 2010; Kraaijeveld et al. 2012).

**Fig. 1.**
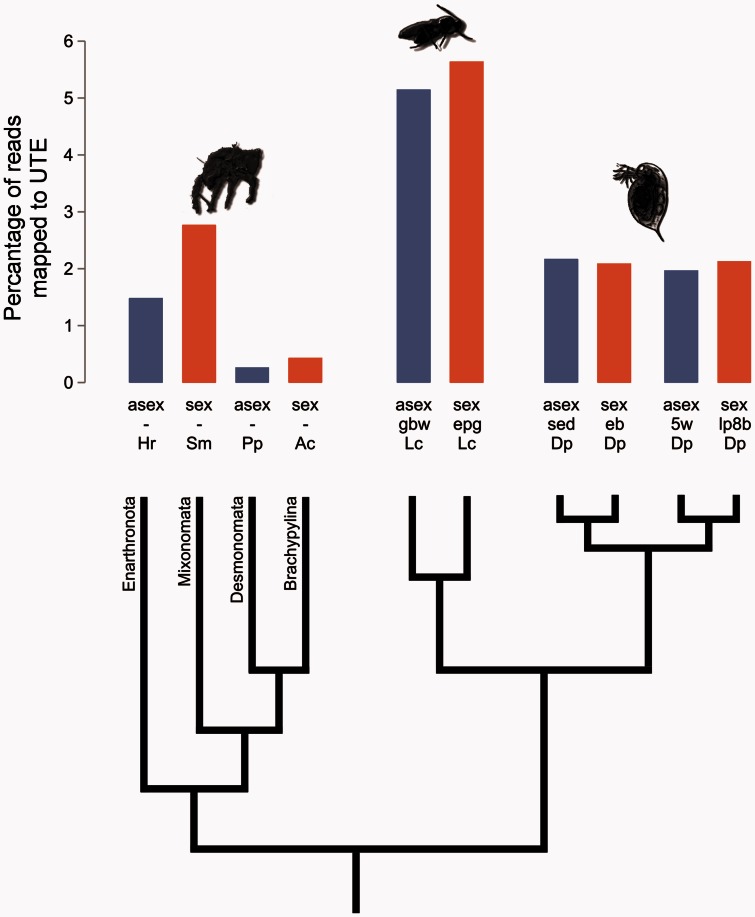
Overall TE loads of independently derived asexual lineages and their sexual relatives from three different arthropod subphyla (Oribatid mites, *Leptopilina* parasitoid wasps, and *Daphnia* water fleas) as estimated by the percentage of reads that mapped to species-specific UTE libraries with 80% homology. Hr, *H. rufulus*; Sm, *S. magnus*; Pp, *P. peltifer*; Lc, *L. clavipes;* Dp, *D. pulex. Leptopilina* wasps comprise one sexual (Gbw) and one asexual (epg) lineage and for *Daphnia* water fleas, two sexual-asexual sister lineages are included sed - eb and 5w - lp8b.

Here, we tested whether asexuality leads to an accumulation of TEs by comparing the genome-wide TE contents between five independently derived asexual animal lineages and their sexual relatives. These five lineages represent three of the four arthropod subphyla (fig. 1), including crustaceans (two asexual lineages of *Daphia pulex* water fleas and their sexual sister lineages), insects (an asexual wasp *Leptopilina clavipes* and its sexual sister lineage), and chelicerates (two asexual and two sexual oribatid mite lineages: *Platynothrus peltifer, Hypochthonius rufulus*, *Steganacarus magnus*,** and *Achipteria coleoptrata*). The five asexual lineages further vary in age, with the *Daphnia* and *Leptopilina* lineages being recently derived from their sexual ancestors (∼22 years and 12,000–43,000 generations for *Daphnia* and *Leptopilina* respectively) and the mites being asexual for at least 10 My (Heethoff et al. 2007; Schaefer et al. 2010; Kraaijeveld et al. 2011; Tucker et al. 2013). Although these age estimates are approximate, given the uncertainty always associated with ages of asexuals (Schurko et al. 2009), the large age gap between the mites and the *Daphnia* and *Leptopilina* lineages allows contrasting TE loads in “old” versus “young” asexuals. To analyze genome-wide TE contents, we used the published genome assemblies for *Daphnia* (Colbourne et al. 2011) and *Leptopilina* (Kraaijeveld et al. submitted) and generated novel assemblies for the four oribatid mite genomes (see Materials and Methods). We quantified the overall content of repetitive DNA and of specific TE families in all ten genomes, and compared the TE contents, as well as their historical activity patterns, between sexual and asexual lineages. Surprisingly, we found extensive variation in TE content among lineages but no differences between sexually versus asexually reproducing lineages. Our results point to fundamental differences in TE dynamics between nonrecombining genome portions and nonrecombining genomes.

## Results

We used two approaches to compare genome-wide TE load between sexual and asexual lineages. First, we compared the complete TE load, without considering putative variation for how different TE classes or (super)families contribute to the load. Different TE types are characterized by different mechanisms through which they spread within a genome, which is known to affect their population dynamics (Burt and Trivers 2006; Pritham 2009). We therefore distinguished different TE (super)families in the second, more detailed approach for testing whether asexual reproduction results in an accumulation of repetitive DNA.

To estimate the complete TE content for each species, we calculated the proportion of genome sequencing reads stemming from TE regions by aligning reads to libraries consisting of the unique portions of TEs (UTE libraries). TE libraries correspond to the list of different TEs in a given genome, whereby each element can be present in one or multiple copies. UTE libraries comprise the same elements but with regions shared between different TE families removed. We used UTE rather than TE libraries to estimate the proportion of TE-containing genome reads because a single read may cluster with multiple elements in a TE library but only with a single element in a UTE library. This would be the case for reads that do not include a unique TE section. (U)TE libraries tend to be lineage specific, with divergent lineages often comprising nonoverlapping lists of elements (Hua-Van et al. 2011). Given the taxon sampling of our study (fig. 1), we therefore used six different UTE libraries: one library for the *Daphnia* lineages, one for the *Leptopilina* lineages, and four libraries for the mite lineages (one library per species). The *Daphnia* and *Leptopilina* UTE libraries were generated from previously published TE libraries (Colbourne et al. 2011; Kraaijeveld et al. submitted), by removing duplicate TE sections; the four mite libraries were generated de novo (see Materials and Methods for details on the libraries and their construction).

There was no evidence for different TE contents in sexual versus asexual lineages. While there is extensive variation in TE content among the three subphyla (*Daphnia*, *Leptopilina* and oribatid mites; *F*_1,6_ = 8.05, *P* = 0.015; fig. 1, supplementary table S1, Supplementary Material online), sexual and asexual lineages did not differ significantly in the percentage of genome reads mapping to the UTE libraries, even after correction for variation among subphyla (*F*_2,7_ = 0.29, *P* = 0.61; fig. 1, supplementary table S1, Supplementary Material online).

**Fig. 2.**
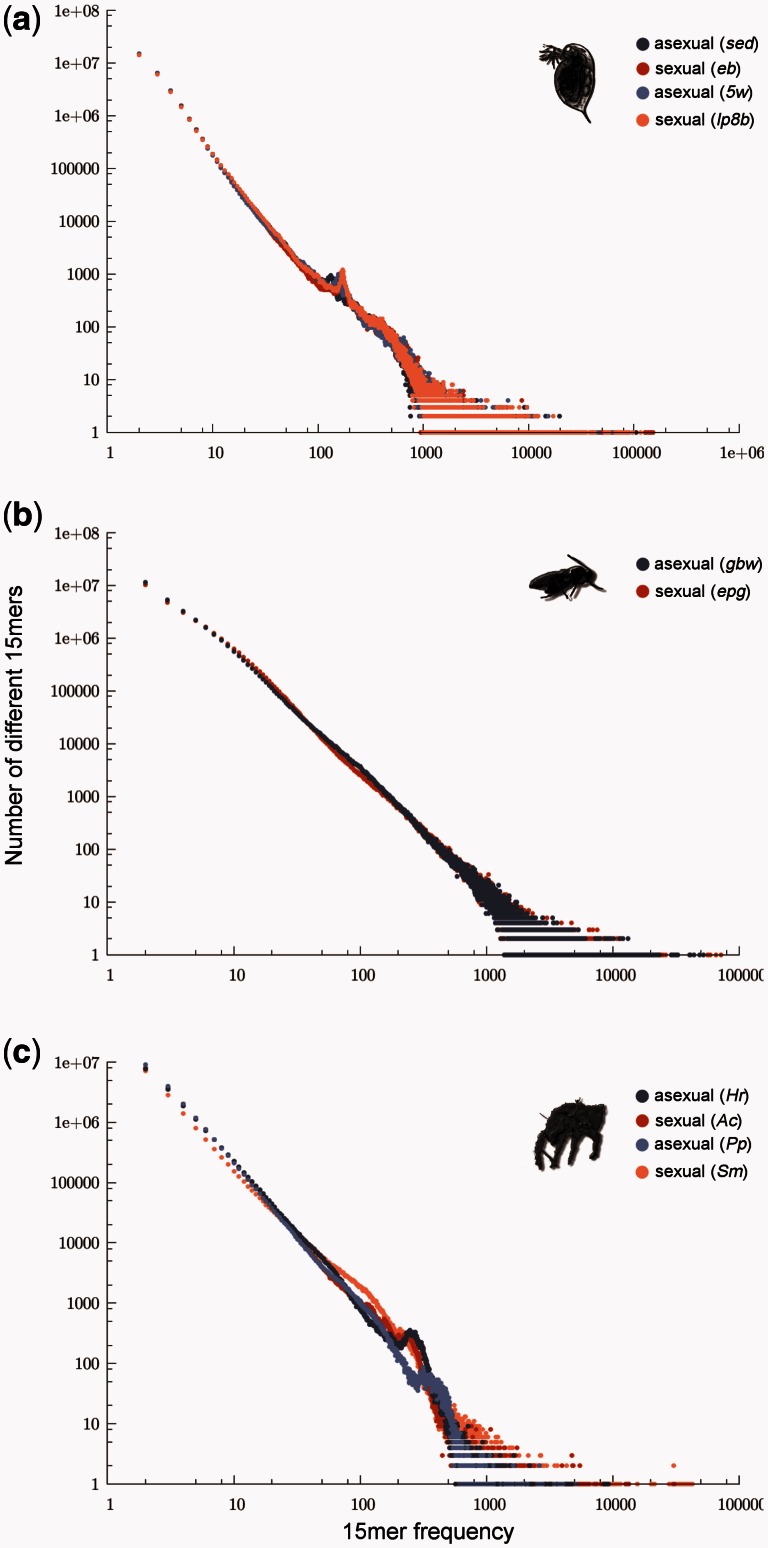
Overall genomic repetitive content of sexual and asexual lineages. Frequency distributions of 15mers in whole-genome read data for (*a*) *Daphnia*, (*b*) *Leptopilina,* and (*c*) oribatid mites. Asexual lineages are depicted in blue and sexual ones in red. Enriched repetitive content in a genome would be indicated by more repeat counts for any divergence class of 15mers—specifically, higher *y*-axis values anywhere along the *x*-axis. Recently expanded TEs generate similar 15mers and would result in a shift of the distribution to the right along the *x*-axis. Long passed TE activity (diverged 15mers) would result in an upward shift of the distribution along the *y*-axis (see also Castoe et al. 2011).

This finding was not caused by incomplete or heterogeneous UTE libraries, as we obtained the same results when quantifying repetitive content with a method from the P-cloud pipeline (Gu et al. 2008) that does not rely on UTE libraries. This method infers whether any sequence portion of specified length (typically 15mers) occurs multiple times within a genome. The rationale is that a particular 15 bp sequence would occur only once in a genome (i.e., the probability of finding two identical, randomly generated 15 bp sequences is very low), except if this sequence was present as multiple copies. Frequency distributions for 15mers are estimated from raw genome sequencing reads, whereby genomes with higher repeat contents would be characterized by higher 15mer counts in the frequency distributions (Castoe et al. 2011). Analogous to our previous TE content comparisons between sexual and asexual lineages, this method did not reveal differences related to reproductive modes. Sexual and asexual *Daphnia* displayed “identical” 15mer frequency distributions (fig. 2*a*), as did sexual and asexual *Leptopilina* lineages (fig. 2*b*). The 15mer frequency distributions of the four oribatid mites differed considerably from each other but irrespective of reproductive mode (fig. 2*c*).

### Load of Different TE Types

Neither of the above analyses of total repetitive DNA load suggested any effect of reproductive mode on genome-wide TE load. However, there are two reasons why comparing total TE loads between sexual and asexual lineages may not be the most appropriate approach for assessing reproductive mode effects on TE accumulation. First, different TE classes differ in the mechanisms underlying their spread in genomes. For example, because some LTR elements specifically target the germline, they are more likely to increase in copy number over time than elements from other TE classes (Lynch 2007). Second, asexual lineages derive from sexual ancestors, such that the specific set, amount, and activity of TEs are dependent on the TEs present at the origin of asexuality. After the evolution of asexuality, new TEs could only be introduced into the genome of an asexual lineage via rare events of horizontal gene transfer. In contrast, TE turnover can be considerable in genomes of sexual lineages, with TE losses and acquisition of new TEs after the split from the asexual sister lineage. Hence, the most pertinent comparison of the effect of reproductive mode on TE dynamics is for the set of elements that are shared via common ancestry between sexual-asexual sister lineages.

For both these reasons, we conducted three additional comparisons between sexual and asexual lineages where we distinguished the contribution of different types of TEs to the complete TE load. First, we quantified the abundance of each specific TE for sexuals and asexuals by estimating coverage per element in the UTE libraries with reads per kilobase mapped (RPKM; supplementary data S1, Supplementary Material online; Tenaillon et al. 2011). In addition to including potential variation among different TEs in their contribution to overall TE loads, this approach includes highly fragmented TE reads or reads that are not assembled at all (which is often the case for repetitive regions in genome assemblies). As expected, different subphyla harbor very different sets of TEs, as revealed by a highly significant interaction between taxonomic group and TE family (*F*_38,1103_ = 7.43, *P* < 0.00001). Reproductive mode did not explain additional variance in content per TE element (*F*_1,1103_ = 0.14, *P* = 0.71).

Second, we compared the load of LTR elements (*Gypsy*, *Copia*, *Pao*) between sexual and asexual lineages. These elements, since they form stable RNA intermediates and may specifically target the germline, are the most likely to generate differences between lineages with different reproductive modes. However, even solely considering these elements, we still found no effect of reproductive mode on LTR-TE load (*F*_1,501_ = 0.15, *P* = 0.70).

**Fig. 3.**
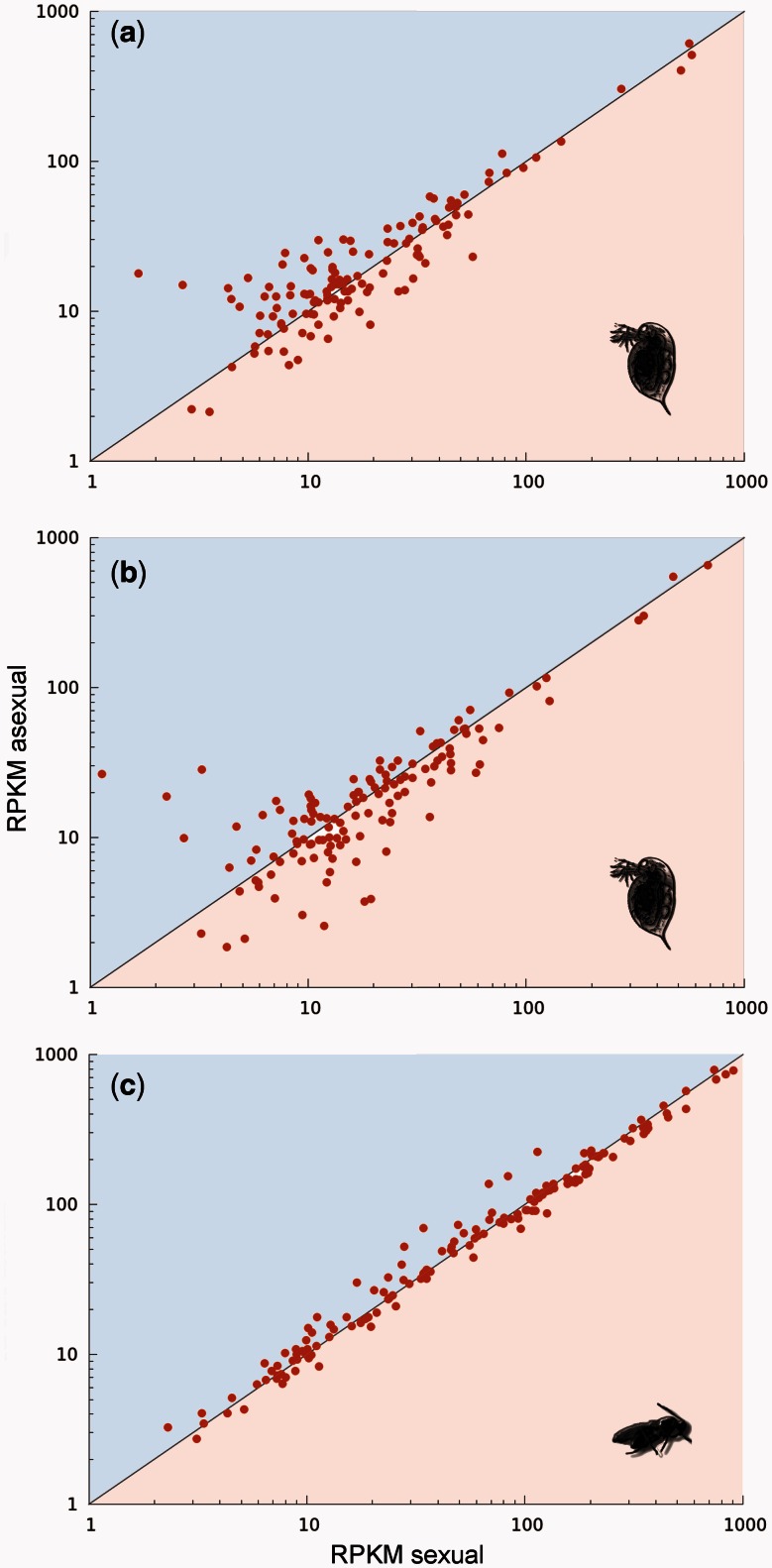
Comparison of load (∼number of copies) per TE family between sexual and asexual sister lineages of *Daphnia* (*a*, *b*) and *Leptopilina* (*c*). Each point represents a specific TE family. Points above the diagonal line indicate more copies of a given family in the asexual lineage and below the line more copies in the sexual lineage (using RPKM values as a proxy for copy number).

Finally, we compared the contribution of different TE types to the complete TE load using only the three asexual lineages (the two *Daphnia* and *Leptopilina*) for which we had a genome for their closest sexual relative at hand (fig. 1). As explained above, TE turn-over in sexual lineages can be substantial after the split of the asexual lineage. Hence, the most powerful analyses to detect reproductive mode effects are comparisons between asexuals and their closest sexual sister lineage and using solely TE types present in both lineages. Again, we found no effect of reproductive mode within lineage pairs on element-specific TE contents (likelihood ratio = 1.16, *P* = 0.28). As in all the previous comparisons, the lack of differences between sexual and asexual lineages was not due to a lack of variation overall. Indeed, some TE elements (*Gypsy* and *Copia*, LTR elements) were enriched in sexuals, others in asexuals (fig. 3, Supplementary fig. S1, Supplementary Material online), indicating strong, lineage-specific TE dynamics but no overall effects of reproductive mode on TE proliferation.

### Repeat Divergence

Given the lack of differences between sexual and asexual lineages in their TE contents, we wanted to develop insights into the mechanisms that could explain why TEs do not accumulate as expected in asexual lineages. For example, TEs might have been inactive or largely lacking at the inception of asexuality. Alternatively, active TEs might have been present originally but become inactive over time.

**Fig. 4.**
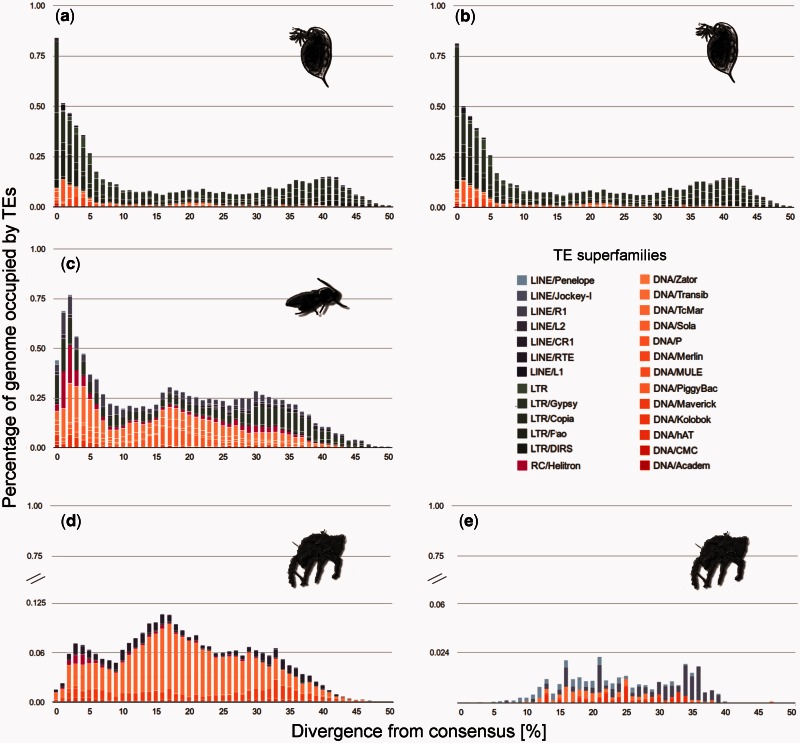
Repeat divergence plots depicting TE activity through time for the most abundant TE families in the genomes of the five asexual lineages (*a*) *Daphnia pulex* (*sed*), (*b*) *Daphnia pulex* (*5w*), (*c*) *Leptopilina clavipes* (*gbw*), (*d*) *Hypochthonius rufulus*, and (*e*) *Platynothrus peltifer.* Element copies with low divergence from the consensus were recently active, whereas TE copies with older activities are more diverged. Note the different *y*-axis scales.

To distinguish these scenarios, we constructed repeat divergence plots (fig. 4) that depict TE activity through time (genetic divergence from TE consensus sequences are used as a proxy for time; Waterston et al. 2002). The rationale is that old copies of a specific TE would be more diverged from each other and from the consensus sequence than a recent copy. In the *Daphnia* asexuals, the most abundant cohorts of TEs were found within 0–6% sequence divergence from the inferred ancestral sequence, indicating a recent burst (fig. 4*a*). In contrast to TEs in *D. pulex*, TE abundance in *L. clavipes* declined at 1% divergence after a burst, indicating a recent decrease in activity (fig. 4*b*)*.* TE bursts are difficult to detect in the asexual mites given the particularly low TE contents in these lineages (fig. 1). Noticeable TE bursts were mostly old (abundance peaks at >10% divergence levels; fig. 4*c* and *d*). Some recent TE bursts (2–10% diverged TEs) occurred in *H. rufulus*, but almost no TEs were found within this range in *P. peltifer.*

## Discussion

In contrast to the expectation that TEs should accumulate in the absence of recombination, our study shows that asexual arthropod lineages are not characterized by higher TE loads than sexual lineages. Although we cannot formally exclude the presence of small but undetectable differences in TE loads between sexuals and asexuals, such differences would be negligible relative to lineage-specific variation not related to reproductive mode but driven by factors such as population sizes or environmental fluctuation (e.g., Hua-Van et al. 2011; Startek et al. 2013). For example, we found 2–6-fold variation in TE loads between different sexual lineages (fig. 1, supplementary fig. S1, Supplementary Material online), a pattern also known for more closely related species than the ones considered here (e.g., 3–25% for different *Drosophila* species; Clark et al. 2007; Kofler et al. 2012). The asexual lineages we analyzed are therefore unlikely to drift towards extinction as a consequence of TE accumulation. Furthermore, the lack of detectable TE load differences between sexual and asexual lineages indicates that dynamics of TE accumulation in nonrecombining genome portions such as sex chromosomes differ greatly from dynamics in nonrecombining genomes.

Our finding that asexual reproduction per se does not result in high TE loads is supported by four previous studies that quantified the load of a small set of specific TEs between a sexual and asexual lineage but found no consistent differences (Rho et al. 2010; Schaack, Choi, et al. 2010; Schaack, Pritham, et al. 2010; Kraaijeveld et al. 2012). Although these studies did not include replicates for sexual and asexual lineages and/or could have missed TE load differences for TEs that were not compared, they corroborate our finding that TE loads are not affected by reproductive modes. The absence of TE accumulation under asexuality further appears to extend to reproduction via self-fertilization or outcrossing in hermaphrodites. Although there is still recombination under self-fertilization, obligately self-fertilizing species are characterized by extensive homozygosity, such that recombination has essentially no effect (Glémin and Galtier 2012). Thus, two comparisons of TE load between outcrossing and selfing plant species revealed either no differences or TE enrichment in the outcrossing species (Slotte et al. 2013; Agren et al. 2015).

What mechanisms could explain the different dynamics of TE accumulation in nonrecombining genome portions as compared to nonrecombining (or effectively nonrecombining) genomes? There are at least three, mutually nonexclusive explanations for the lack of TE accumulation in nonrecombining genomes. First, TE dynamics are likely to be influenced by molecular mechanisms, and some mechanisms could differ between genomes of sexual and asexual lineages. For example, changes in methylation patterns upon the transition to asexuality could constrain TE proliferation in asexuals, or TEs could also be lost as a consequence of elevated rates of gene conversion. Such proximate mechanisms could explain why asexuals do not harbor more TEs than sexuals even though selection would be more efficient at removing TEs in sexuals than asexuals.

Second, it is possible that new asexual animal lineages arise frequently from sexual ancestors but that TE accumulation would drive the majority of them extinct. Extinction would occur so rapidly that their transient existence in natural populations would never be recognized. Under this scenario, the analyzed asexual animal lineages would represent the small fraction of asexuals that inherited only few or inactive TEs from their sexual ancestors. Although the low TE content we found in all (sexual and asexual) lineages here is consistent with this scenario, there are at least three reasons why we consider it unlikely. First, the asexual *D. pulex* lineages are recently derived asexuals, with age estimates of only 22 years (Tucker et al. 2013). Second, there are highly successful asexual animals with much higher TE loads (e.g., 36% in the asexual nematode *Meloidogyne incognita;* Abad et al. 2008). Finally, very frequent transitions from sexual to asexual reproduction are quite unlikely in most taxa, given such transitions are hampered by a number of genetic and developmental constraints (Engelstädter 2008; Neiman et al. 2014).

The third possible explanation for the lack of TE accumulation in nonrecombining genomes is that upon the transition to asexuality, benign (self-regulated) elements should be selectively favored (Charlesworth and Langley 1986). TEs can be considered as selfish intragenomic parasites, given they generate additional copies of themselves while (usually) generating negative fitness in their hosts (Hickey 1982; Burt and Trivers 2006). As long as hosts reproduce sexually, recombination and genetic exchange between hosts uncouples the fate of a TE and an individual host genome. As a consequence, selection favors TEs that efficiently transmit themselves between hosts, independently of potential fitness consequences for the hosts (Hickey 1982). In contrast, obligate asexual reproduction couples the fate of TEs and their hosts, resulting in selection for TEs that do not induce negative fitness effects in their hosts. Such selection could favor, for example, low transposition rates and site-specific transposition to genomically “safe sites” (i.e., nonfunctional regions) and is expected to result in a decrease of TE activity over time (Charlesworth and Langley 1986; Burt and Trivers 2006). For example, such self-regulation was suggested to explain the spread of an experimentally introduced TE in sexual but not asexual strains of yeast (Zeyl et al. 1996). Consistent with this expectation, TE repeat divergence indicated that the genomes of the three young asexuals (two *D. pulex* and *L. clavipes*) experienced recent TE activity and that this activity stopped in the somewhat older *Leptopilina* lineage (fig. 4*a* and *b*). Asexual oribatid mite TEs showed no signs of recent activity (fig. 4*c* and *d*) a pattern of particular interest since *H. rufulus* and *P. peltifer* have been asexual over evolutionary timescales (Heethoff et al. 2007; Schaefer et al. 2010).

In conclusion, we conducted several detailed analyses of whole-genome TE content in five independently derived asexual animals and their sexual relatives but found no support for the prediction that asexuality results in the accumulation of TEs. This finding contrasts with the accruing evidence for the accumulation of other types of deleterious mutations in asexuals (Paland and Lynch 2006; Neiman et al. 2010; Henry et al. 2012; Hollister et al. 2015; but see Tucker et al. 2013) and the enrichment of TEs in nonrecombining genome portions of sexual species (Lynch and Blanchard 1998; Bachtrog 2013; Schwander et al. 2014; Leung et al. 2015). Inferring whether TE accumulation in asexuals is constrained by proximate mechanisms, or whether TEs in asexuals tend to be self-regulated, is a challenge for future studies. Our results indicate that TEs are unlikely to drive decay or extinction of extant asexual lineages and suggest that genome-wide lack of recombination generates different TE dynamics than lack of recombination in isolated genome portions.

## Materials and Methods

### Genomic Data

Genomic next-generation sequencing read data of two sexual (*eb-1*, *lp8b-6*) and two asexual (*sed-2*, *5w-2*) lineages of *D. pulex* were retrieved from (Tucker et al. 2013). For the asexual lineages, assemblies were constructed with mosaik using default settings (Lee et al. 2014) in a reference-guided fashion, using the available sexual *D. pulex* genome (Colbourne et al. 2011; Acc. no. ACJG00000000.1). For *L. clavipes*, read data were obtained from (Kraaijeveld et al. 2012) for a sexual (*epg*) and an asexual (*gbw*) lineage. The genome assembly of the asexual lineage is also available (Acc. no. PRJNA84205; Kraaijeveld et al. submitted). For the four oribatid mites, we generated de novo draft genome assemblies from a pool of 50 individuals for each species. For mite collection, litter and organic soil layers were gathered from forests near Göttingen, Germany. Mites were separated from litter using gradient heat extraction (Kempson et al. 1963) and collected in water. Living animals were identified following Weigmann (2006). Prior to DNA extraction, individuals were starved for ten days and cleaned with water and ethanol to minimize contamination with nonmite DNA. Genomic DNA was extracted using the DNeasy Blood and Tissue kit (Qiagen). Paired-end Illumina sequencing was done at the Leiden Genome Technology Center (LGTC; Leiden, The Netherlands) with a single insert size of 300 bp. *Steganacarus magnus* and *P. peltifer* were run on one lane of a GaIIx system generating 75 bp paired-end reads. *Achipteria coleoptrata* and *H. rufulus* were run on one lane of a HiSeq2000 system, producing 100-bp paired-end reads. Raw reads of all lineages were quality filtered and duplicates were removed using Trimmomatic and Fastx-Toolkit (Hannon Laboratory 2010; Bolger et al. 2014). Mite genomes were assembled using Abyss (for *S. magnus* and *P. peltifer*) and Platanus (for *A. coleoptrata* and *H. rufulus*) with default parameters (Simpson et al. 2009; Kajitani et al. 2014). Two different assemblers were used due to the different data produced by the two sequencing methods. Assembly of oribatid mite genomes resulted in fragmented draft genomes with N50 metrics ranging from 1.6 kb to 7.4 kb for scaffolds bigger than 200 bp (supplementary table S2, Supplementary Material online; PRJNA280488).

### UTE Libraries

Species-specific TE libraries were downloaded from RepBase (Jurka et al. 2005) for *D. pulex* and obtained from Kraaijeveld et al. (unpublished data) for *Leptopilina.* For oribatid mites, de novo repeat detection was done separately for each of the four genomes. We used both assembled data and quality filtered raw reads with self-homology and overrepresented sequence detection approaches to obtain TEs with low copy number as well as potentially excluded TEs in the assemblies, running RepeatModeler (Smit and Hubley 2011) and Tedna (Zytnicki et al. 2014; with 45–60 million reads per species and the -t option set to 30). These TE detection methods construct TE families consensus sequences based on 80% repeat homology. Output sequences larger than 500 nt were clustered with 95% identity threshold using uclust (Edgar 2010) with the centroid option to join fragments and reduce redundancy. To identify TEs, REPCLASS (Feschotte et al. 2009) and homology searches were run using RepeatMasker (Smit et al. 1996), tBLASTx, and BLASTn against RepBase (Jurka et al. 2005) and nonredundant NCBI entries (keywords: retrotransposon, transposase, reverse transcriptase, transposon, TE; *e* value > 1e-30). Sequences were discarded if all annotation methods regarded library entries as “unknown” (56–79% of the sequences) and remaining entries were collected in “draft TE libraries.” Entries in the draft TE library with protein homology in less than 40% of the sequence length or ambiguous annotation (i.e., two likely elements) were validated with the online version of Censor (Kohany et al. 2006) against RepBase. As a final check, entries in the draft TE libraries were blasted against all NCBI entries to remove sequences with high similarity to non-TE entries, resulting in the final TE library. All library TE headers were reformatted to match RepeatMasker naming standards. Resulting TE libraries contained numbered elements classified to super-family level (supplementary table S3, Supplementary Material online).

All TE libraries were then reduced to UTE libraries following Tenaillon et al. (2011). The species-specific, clustered TE libraries were split into 104 bp fragments and mapped back to the original library with 80% homology (the TE family classification criterion). If elements or portions of elements were covered more than once, duplicate copies were removed, and only one copy was kept in the UTE library. Per library, a maximum of 2% duplicate sequences were removed.

Genome-specific, unique TEs cannot be detected if sequencing reads of a specific genome are aligned to UTE libraries generated for a different genome. To avoid such nondetection of unique TEs, we generated genome-specific UTE libraries for each of the four mite species. Genome-specific libraries could not be generated for *Leptopilina* and *Daphnia* as we used previously published genome data for these two species. The TE libraries in *Leptopilina* are derived from the asexual strain, hence we might not detect potential TEs specific to the sexual strain. Importantly, however, the presence of undetected TEs specific to the sexual strain would only further strengthen our findings. For *D. pulex*, TE libraries are based on a sexual genome, and unique TEs in the asexual genomes could indeed remain undetected. However, the presence of undetected TEs in the asexual *D. pulex* genomes is highly unlikely for two reasons. First, when mapping reads from all four *D. pulex* genomes against the *D. pulex* TE library, each TE from the library is present in all four genomes (no element in the library is specific to the sexual lineage used for generating the reference genome; data not shown). Second, the *D. pulex* lineages are of very recent origin, making it unlikely that asexuals acquired new TEs through rare horizontal transfer or that both sexual lineages lost all copies of a TE family during this time.

### TE Quantification

For TE quantification, we followed Tenaillon et al. (2011). Reads for each lineage were mapped against the respective UTE using ssaha2 (Ning et al. 2001) with best hit option and homology of 80%, according to TE family classification criteria. Reads aligning for less than 30 bp were discarded. For complete TE abundances, the fraction of reads that mapped to the UTE compared to total reads was calculated for each lineage. For each TE entry in the UTEs, RPKM was calculated to assess the relative TE load of each lineage, accounting for differences in sequence fragment length with (RPKM_entry = (reads_mapped_entry/((length_entry/1,000) * (total_mapped_reads/1,000,000))) (supplementary data S1, Supplementary Material online).

### Repetitive Content

Most methods for de novo TE detection depend on databases of known elements (i.e., TE or UTE libraries) and might fail to identify unknown, highly fragmented and ancient TEs. Therefore, to get general insights into genomic repetitive content of each lineage or species, 15mer frequencies using 1-fold coverage, quality trimmed read data were calculated following Castoe et al. (2011) with P-clouds C10 settings (Gu et al. 2008). For lineages of *Daphnia* and *Leptopilina*, the 1-fold coverage amount of read data was used. For mites, the 1-fold read data equivalent of *A. coleoptrata* was extracted for each species, as genome sizes vary.

### TE Activity

To assess TE activity through time within a given genome, repeat divergences were computed by calculating the Kimura-CpG-corrected divergence between the consensus sequence (as constructed in the TE libraries) of each specific TE and all its copies in the genomes. First, TEs in the assemblies were identified using RepeatMasker with the sensitive option. TE divergences were then computed using the script calcDivergenceFromAlign.pl and plotted with createRepeatLandscape.pl implemented in RepeatMasker.

### Statistics

All statistical analyses were conducted in R version 3.1.3 (R Core Team 2015). For overall TE abundance comparisons, the proportion of reads that map to TEs was compared between sexual and asexual taxa in a GLM with a quasi-binomial error distribution. For RPKM comparisons, RPKM values were log transformed and analyzed via linear models using the R library nlme (Pinheiro et al. 2015). For each comparison, the change in deviance explained when dropping the effect of reproductive mode (and/or the effect of subphylum) was evaluated using an *F* test. Complete model results are available from the authors upon request.

## Supplementary Material

Supplementary figure S1, data S1, and tables S1–S3 are available at *Molecular Biology and Evolution* online (http://www.mbe.oxfordjournals.org/).

Supplementary Data
