# Supplementary material for: No Accumulation of Transposable Elements in Asexual Arthropods
Source: Mol Biol Evol. 2015 Nov 11;33(3):697–706. doi: 10.1093/molbev/msv261 (PMC4760076; doi:10.1093/molbev/msv261)
Supplement: Supplementary Data [file supp_33_3_697__index.html]

No Accumulation of Transposable Elements in Asexual Arthropods — No Accumulation of Transposable Elements in Asexual Arthropods — Supplementary Data 

# No Accumulation of Transposable Elements in Asexual Arthropods

## Supplementary Data

files

- Supplementary Data - xls file
- Supplementary Data - pdf file
