# Supplementary material for: No Accumulation of Transposable Elements in Asexual Arthropods
Source: Mol Biol Evol. 2015 Nov 11;33(3):697–706. doi: 10.1093/molbev/msv261 (PMC4760076; doi:10.1093/molbev/msv261)
Supplement: Supplementary Data [file supp_msv261_Supplementary_Information.pdf]

Supplementary Information

No accumulation of transposable elements in asexual animals

Jens Bast[1], Ina Schaefer[2], Tanja Schwander[1], Mark Maraun[2], Stefan Scheu[2], Ken Kraaijeveld[3,4]*

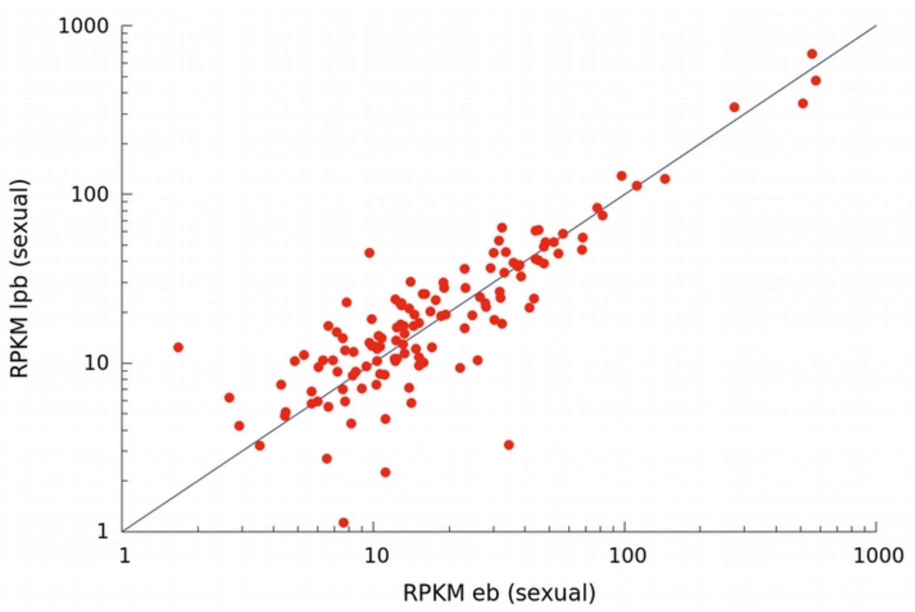

Supplementary Figure 1: Comparison of load (RPKM as proxy) per TE family between sexual lineages of *Daphnia*. Each point represents a specific element family in the TE library.

Supplementary Table 1: Complete TE content in sexual and asexual lineages calculated from reads mapping to the species-specific UTEs with 80% alignment homology. Ori: Oribatid mites, Lc: *Leptopilina clavipes*, Dp: *Daphnia* pulex, Ac: *Achipteria coleoptrata*, Hr: *Hypochthonius rufulus*, Sm: *Steganacarus magnus*, Pp: *Platynothrus peltifer*

tax_group	Lineage	Mode	Total_reads	Mapped_reads	Percentage
Ori	Ac	sex	68023704	293628	0.43
Ori	Hr	asex	62628353	929356	1.48
Ori	Sm	sex	54559873	1510343	2.77
Ori	Pp	asex	54940089	144433	0.26
Lc	epg	sex	45085169	2540611	5.64
Lc	gbw	asex	47605995	2450454	5.15
Dp	eb	sex	28650165	599742	2.09
Dp	sed	asex	29643143	644050	2.17
Dp	lp8b	sex	28891907	615635	2.13
Dp	5w	asex	29001545	571543	1.97

Supplementary Table 2: Sequencing data produced for four oribatid mite species and assembly statistics for contigs > 200 bp. Ac: *Achipteria coleoptrata*, Hr: *Hypochthonius rufulus*, Sm: *Steganacarus magnus*, Pp: *Platynothrus peltifer*

Species		**Ac**	**Hr**	**Sm**	**Pp**
	Mode of reproduction	sexual	asexual	sexual	asexual
Sequencing data	Sequencing system	HiSeq2000	HiSeq2000	GAIIx	GAIIx
	Insert size [bp]	300	300	300	300
	Read-length [bp]	100	100	75	75
	Number filtered reads	290119558	274538142	58096450	57360134
	Coverage	~176x	~120x	~16x	~19x
Assemblies	Assembler	Platanus	Platanus	Abyss	Abyss
	Number contigs	56361	140559	101559	105693
	N80 [bp]	1440	905	609	561
	N50 [bp]	7410	4184	2446	1557
	Max [bp]	158988	132029	53110	34640
	Sum [bp]	87.47e6	171.8e6	112.6e6	99.84e6

Supplementary Table 3: Transposable element library construction and comparison of TE entries > 500bp. Ac: *Achipteria coleoptrata*, Hr: *Hypochthonius rufulus*, Sm: *Steganacarus magnus*, Pp: *Platynothrus peltifer*, Lc: *Leptopilina clavipes*, Dp: *Daphnia pulex*

Species		**Ac**	**Hr**	**Sm**	**Pp**	**Lc**	**Dp**
Total repeats	RepeatModeler	289	600	235	110	-	-
	Tedna	266	218	207	95	-	-
	Combined after UCLUST	450	807	435	205	-	-
Classified TEs	Number of TE sequences	74	117	153	16	162	229
	Number of super-families	10	13	11	7	25	22
	Mean length [bp]	1016	848	999	1176	1886	3108
	Combined length [bp]	75200	99260	152965	18828	305641	711853
	Maximum size [bp]	4838	2505	4665	3899	11311	18820
